# Folic Acid Supplement Intake Before Pregnancy With and Without Educational Intervention: A Cross-Sectional Study in Japan

**DOI:** 10.7759/cureus.87959

**Published:** 2025-07-15

**Authors:** Miho Morioka, Yuki Kataoka, Shunsuke Taito

**Affiliations:** 1 Department of Life and Culture Studies, Wakayama Shin-Ai Women's Junior College, Wakayama, JPN; 2 Department of Internal Medicine, Kyoto Min-iren Asukai Hospital, Kyoto, JPN; 3 Department of Systematic Reviewers, Scientific Research WorkS Peer Support Group (SRWS-PSG), Osaka, JPN; 4 Department of Healthcare Epidemiology, School of Public Health, Kyoto University Graduate School of Medicine, Kyoto, JPN; 5 Department of International and Community Oral Health, Tohoku University Graduate School of Dentistry, Sendai, JPN; 6 Department of Clinical Practice and Support, Hiroshima University Hospital, Hiroshima, JPN

**Keywords:** before pregnancy, folic acid, health education, neural tube defects (ntds), nutrition education, supplement intake

## Abstract

Background

Although folic acid supplementation during pregnancy has been recommended for over 20 years, awareness remains insufficient. This study aimed to investigate whether graduates who received folic acid education at a dietitian training school intended to take supplements prior to future pregnancies and to examine the methods of information dissemination.

Methods

This cross-sectional study targeted graduates of the Department of Lifestyle and Culture at Wakayama Shin-Ai Women’s Junior College, Wakayama, Japan. Data were collected via a questionnaire administered through Google Forms (Google LLC, Mountain View, CA, USA). The primary outcome was the intention to take folic acid supplements before pregnancy, and the main exposure was having received folic acid nutrition education during student years. A logistic regression model was used to assess associated factors.

Results

A total of 1,109 graduates (532 from the Life Culture major and 577 from the Food and Nutrition major) were eligible. Forty-eight responses were received, with 47 considered valid (response rate: 4.2%). Among the valid responses, 24 participants (51%) indicated they would take folic acid supplements before future pregnancies, while 23 (49%) reported they would not. A Food and Nutrition major was strongly associated with a higher intention to take folic acid supplements (OR: 17.69, 95% CI: 2.91-343.83). Awareness of neural tube defects (NTDs; OR: 3.81, 95% CI: 1.17-13.49) and access to folic acid-related information (OR: 7.20, 95% CI: 2.07-28.94) were also positively associated with this intention. Furthermore, participants who recalled learning about folic acid during junior college were more likely to intend to take supplements (OR: 6.50, 95% CI: 1.80-28.14).

Conclusions

Having a Food and Nutrition major, awareness of NTDs, access to folic acid information, and prior education about folic acid were positively associated with the intention to take folic acid supplements before pregnancy. However, due to the very low response rate, the multivariate findings were unstable and exploratory. Therefore, interpretation was limited to univariate results. Further prospective cohort studies with larger sample sizes are needed to confirm these associations.

## Introduction

Folic acid plays a critical role in cell growth. Neural tube defects (NTDs) are congenital malformations that occur when the neural tube fails to close properly, typically around 28 days after conception. Numerous intervention studies have demonstrated that folic acid supplementation before and during early pregnancy significantly reduces the risk of NTDs [1]. Several studies have also emphasized the importance of initiating folic acid supplementation prior to conception [2-7].

However, awareness of the recommendation to take folic acid to prevent NTDs remains insufficient, particularly among women who have never been pregnant. This has been highlighted in the “Survey report on appropriate nutrition and dietary habits for pregnancy and childbirth” [8] and the “Final recommendation statement: Folic acid supplementation to prevent neural tube defects - preventive medication” (August 1, 2023) [9].

Although folic acid supplementation during pregnancy has been recommended for over 20 years [10], public awareness of this guidance continues to be inadequate. In light of this, the present study aimed to assess whether graduates of dietitian training schools - who received education on folic acid during their studies - intend to take folic acid supplements during their own pregnancies.

This study addresses the following research question: What factors are associated with the intention to take folic acid supplements before pregnancy among women of reproductive age?

Based on previous findings, we hypothesized that awareness of NTDs, access to information about folic acid, and relevant educational background would be positively associated with the intention to take folic acid supplements. Therefore, the objective of this study was to examine these associations through a cross-sectional survey conducted among junior college graduates.

## Materials and methods

Study design

This study was conducted in accordance with the STrengthening the Reporting of OBservational Studies in Epidemiology (STROBE) guidelines [11]. A cross-sectional survey design was employed.

Questionnaire development

The questionnaire was developed in two stages: (1) a review of previous studies and selection of items by the authors and (2) an evaluation of content validity. For stage (1), a thorough review of both domestic and international literature on folic acid intake during pregnancy was conducted. Variables frequently reported and aligned with the purpose of this study were identified through discussion and consensus among the authors [8]. For stage (2), the research team - comprising one member with over 20 years of experience in dietitian education and two with expertise in clinical epidemiology - reviewed and cross-evaluated the questionnaire items and terminology based on their professional judgment. The draft questionnaire was then reviewed by ten individuals (including a faculty member from the junior college affiliated with the first author) to assess clarity and content validity. Based on their feedback, unclear items were revised, and the final version of the questionnaire was established. Informed consent was obtained explicitly, with participants confirming consent by checking a confirmation box after reading the information statement.

Setting

The study targeted graduates from the Department of Life Culture at Wakayama Shin-Ai Women’s Junior College who completed their studies between March 2006 and March 2023. An article in the annual alumni newsletter invited participation from graduates of both the Life Culture major and the Food and Nutrition major. In the Food and Nutrition major, students received formal education on the importance of folic acid supplementation before pregnancy. Participants accessed the questionnaire via a QR code provided in the alumni publication. Data collection was conducted between September and December 2023.

Participants

All participants provided informed consent and voluntarily completed an anonymous questionnaire.

Variables

The primary outcome of this study was the intention to take folic acid supplements before future pregnancies. We examined seven factors as potential predictors of this intention: (1) major (Life Culture or Food and Nutrition); (2) pregnancy experience; (3) awareness of NTDs; (4) awareness of the role of folic acid in reducing the risk of NTDs; (5) access to information about folic acid; (6) recollection of learning about folic acid during junior college; and (7) whether the participant had shared information with others about the necessity of taking folic acid supplements before pregnancy. The relevant survey items were as follows: Question 3 (major: Life Culture or Food and Nutrition), Question 4 (pregnancy experience), Question 14 (awareness of NTDs), Question 15 (awareness of the preventive role of folic acid), Question 16 (access to information about folic acid), Question 18 (recollection of learning about folic acid during junior college), Question 19 (sharing folic acid information with others), and Question 20 (intention to take folic acid supplements before future pregnancies). The full questionnaire is provided in the Appendices section.

Sample size

The estimated target population included approximately 1,100 alumni association members. A 30% response rate was expected, with a target of around 330 completed responses. Based on historical enrollment data (approximately 50 students per year in the Food and Nutrition major and 40 in the Life Culture major), an even distribution of responses between the two majors was anticipated.

Statistical methods

Descriptive statistics were reported as counts and percentages for categorical variables. For univariate analysis, logistic regression was used to assess differences based on demographic characteristics and folic acid awareness. Multivariate logistic regression was performed to analyze associations between the seven selected variables and the likelihood of intending to take folic acid supplements before pregnancy. All analyses were conducted using RStudio.

Ethical approval

This study was approved by the Research Ethics Committee of Wakayama Shin-Ai Women’s Junior College on October 26, 2022.

## Results

Participants

Of the 1,109 eligible graduates from the Department of Life Culture - comprising 532 from the Life Culture major and 577 from the Food and Nutrition major - a total of 48 responses were received, with 47 deemed valid, resulting in a response rate of 4.2%.

Descriptive data

Among the 47 valid respondents, 24 (51%) expressed an intention to take folic acid supplements before future pregnancies, while 23 (49%) indicated they would not (Table 1).

**Table 1:** Presence or absence of folic acid supplement intake before future pregnancies Percentages represent column percentages based on the number of respondents in each group (“Taking” and “Not taking”). NTD, neural tube defect

Characteristic	Taking folic acid supplements before future pregnancies (N = 24)	Not taking folic acid supplements before future pregnancies (N = 23)
Major in Dietetics	23 (96%)	13 (57%)
Major in Life and Culture	1 (4%)	10 (43%)
Experienced pregnancy	8 (33%)	3 (13%)
No experience of pregnancy	16 (67%)	20 (87%)
Aware of NTDs	15 (63%)	7 (30%)
Not aware of NTDs	9 (38%)	16 (70%)
Aware of the role of folic acid in reducing the risk of NTDs	9 (38%)	4 (17%)
Not aware of the role of folic acid in reducing the risk of NTDs	15 (63%)	19 (83%)
Access to information about folic acid	16 (67%)	5 (22%)
No access to information about folic acid	8 (33%)	18 (78%)
Recalls learning about folic acid during junior college	20 (83%)	10 (43%)
Does not recall learning about folic acid during junior college	4 (17%)	13 (57%)
Has provided information to others about the necessity of folic acid supplements	11 (46%)	0 (0%)
Has not provided information to others about the necessity of folic acid supplements	13 (54%)	23 (100%)

Univariate analysis

Table 2 presents the results of the univariate analysis. Graduating with a major in Food and Nutrition was strongly associated with a higher intention to take folic acid supplements before future pregnancies (OR: 17.69, 95% CI: 2.91-343.83), suggesting that academic major may significantly influence this behavior. Pregnancy experience showed a weak or non-significant association with the outcome (OR: 3.33, 95% CI: 0.82-17.17). Both awareness of NTDs (OR: 3.81, 95% CI: 1.17-13.49) and access to information about folic acid (OR: 7.20, 95% CI: 2.07-28.94) were positively associated with the intention to take supplements. Similarly, recalling having learned about folic acid during junior college was also linked to a higher intention to take supplements (OR: 6.50, 95% CI: 1.80-28.14). However, the effect of having provided information to others about the necessity of folic acid supplementation before pregnancy was highly uncertain (OR: 204,609,403.31, 95% CI: 0.00-NA) and should be interpreted with caution due to the wide and undefined CI.

**Table 2:** Factors associated with taking folic acid supplements before future pregnancies NA, not applicable (due to the limited sample size, model estimation was unstable); NTD, neural tube defect

Characteristic	Univariate OR (95% CI)	Multivariate OR (95% CI)
Major	17.69 (2.91-343.83)	2.90 (0.06-203.60)
Experienced pregnancy	3.33 (0.82-17.17)	62,138,955.22 (0.00-NA)
Awareness of NTDs	3.81 (1.17-13.49)	0.70 (0.08-5.99)
Awareness of reducing the risk of NTDs	2.85 (0.77-12.24)	0.39 (0.03-3.73)
Access to information about folic acid	7.20 (2.07-28.94)	2.11 (0.34-15.28)
Remembering learning it in junior college	6.50 (1.80-28.14)	126,692,990.78 (0.00-NA)
Providing information to others about the necessity of folic acid supplements before pregnancy	204,609,403.31 (0.00-NA)	187,205,712.46 (0.00-NA)

Multivariate analysis

Table 2 presents the results of the multivariate logistic regression analysis. Graduating with a major in Food and Nutrition showed little to no effect on the outcome (OR: 2.90, 95% CI: 0.06-203.60). The effect of pregnancy experience on the outcome was highly uncertain (OR: 62,138,955.22, 95% CI: 0.00-NA). Awareness of NTDs (OR: 0.70, 95% CI: 0.08-5.99) and awareness of strategies to reduce the risk of NTDs (OR: 0.39, 95% CI: 0.03-3.73) both showed weak or no association with the outcome. Access to information about folic acid was weakly positively associated or not associated with the outcome (OR: 2.11, 95% CI: 0.34-15.28). The effects of recalling having learned about folic acid during junior college (OR: 126,692,990.78, 95% CI: 0.00-NA) and providing information to others about the necessity of folic acid intake before pregnancy (OR: 187,205,712.46, 95% CI: 0.00-NA) were also highly uncertain due to wide and undefined CIs.

## Discussion

We conducted a survey among graduates of Wakayama Shin-Ai Women’s Junior College to investigate the factors influencing the intention to take folic acid supplements before pregnancy. The response rate was 4.2% (47 valid responses out of 1,109), revealing several noteworthy correlations. Graduates from the Dietetics major were more likely to intend to take folic acid supplements compared to those from the Life Culture major. Additionally, recalling folic acid education during junior college, awareness of NTDs, and access to information about folic acid were all associated with the intention to take supplements.

Pal et al. reported that most participants in their study had insufficient knowledge and awareness of folic acid supplementation, emphasizing the need for health education and information, education, and communication activities to improve folic acid knowledge among women of reproductive age [12]. Similarly, Ummair et al. examined compliance with iron and folic acid supplementation among pregnant women attending antenatal care and found low adherence rates, highlighting a significant public health concern [13]. Furthermore, Ledet et al., in their review of folic acid knowledge across five countries, concluded that all five countries must better promote the importance of folic acid in preventing NTDs and the need for supplementation before and during pregnancy [14].

In line with these concerns, the Dietary Guidelines for Pregnant and Lactating Women were revised in March 2021 to the Dietary Guidelines for Pregnant and Lactating Women Starting Before Pregnancy, stressing the importance of building a healthy body and establishing proper eating habits before pregnancy begins [15].

In our study, despite receiving coursework on folic acid, only 64% (23 out of 36) of respondents from the Food and Nutrition major reported an intention to take folic acid supplements before pregnancy. This difference in intention between Dietetics and Life Culture graduates highlights the potential impact of specialized education. In the Dietetics major, the importance and method of folic acid supplementation are systematically taught, likely enhancing students’ understanding and encouraging adoption of such practices. Existing literature supports this, as nutritional education has been shown to promote healthier behaviors [16]. Therefore, expanding education on folic acid may help increase supplementation intentions. However, revising the curriculum for non-Dietetics majors may be difficult due to different academic focuses and pre-existing course requirements.

Given these constraints, providing ongoing information through alternative means - such as posters and educational materials - could be a practical solution. Awareness of folic acid recommendations to prevent NTDs among women with no pregnancy experience remains insufficient, as noted in the FY 2018 Research Project on Child and Childcare Support Promotion [8]. Our findings suggest that folic acid education can play an important role in shaping young women’s intentions to take folic acid supplements before pregnancy, particularly by increasing awareness of NTDs and improving access to relevant information.

This study has several limitations. Most notably, the response rate was only 4.2%, raising concerns about both internal and external validity. Selection bias may have occurred, particularly if respondents were more health-conscious or already aware of folic acid recommendations, potentially leading to either an overestimation or underestimation of associations. The distribution of majors was also imbalanced, with 77% from the Dietetics major and only 23% from the Life Culture major. Additionally, the small sample size limited the ability to conduct a robust multilevel multivariable logistic regression analysis. Furthermore, some subgroups had very few outcome events, which resulted in extremely large or unstable odds ratios and may have introduced estimation bias.

## Conclusions

A major in food and nutrition, awareness of NTDs, access to information about folic acid, and educational experiences in junior college were positively associated with the intention to take folic acid supplements before pregnancy. However, due to the extremely low response rate, the multivariate results were unstable and should be considered exploratory. As a result, interpretation was limited to univariate findings. Further prospective cohort studies with larger sample sizes are necessary to confirm these associations.

## Appendices

Questionnaire on the intake of folic acid supplements before pregnancy based on folic acid education

Dear Alumni,

Thank you for your continued support and understanding of our educational and research activities. My name is Miho Morioka. I belong to the Department of Life and Culture Studies Major in Dietetics. I am in charge of subjects related to "Nutrition Education" and conduct research on nutrition education across various life stages. We are currently conducting a research study on dietary habits before pregnancy.

This study aims to investigate whether graduates took folic acid supplements during their pregnancies and to evaluate the effectiveness of information provision, considering that the supplementation of folic acid during pregnancy has been recommended for more than 20 years but is still not widely recognized.

This survey has been reviewed and approved by the Ethics Committee of Wakayama Shin-Ai Women's Junior College. Participation in this survey is voluntary, and there will be no disadvantages for those who choose not to participate. The survey will be conducted online and will be anonymous. You are not required to provide personal information such as your name or address. The results of the anonymous survey will be used as research data. Please note that once you have submitted your responses, it will not be possible to withdraw them as the responses will be anonymous and the researchers will not be able to identify who provided them.

We kindly ask for your cooperation in this survey.

Target Audience: Graduates of the Department of Life Culture (Life Culture Major and Food and Nutrition Major) at Wakayama Shin-Ai Women's Junior College.

Time Required: 3 minutes

Response Deadline: Thursday, November 30, 2023

Purpose of Use: Publication (academic papers and conference presentations)

Miho Morioka, Associate Professor, Department of Life Culture, Food and Nutrition Major, Wakayama Shin-Ai Women's Junior College

Please proceed only if you are willing to participate in this survey. If you are willing to cooperate, please check the confirmation box below.

☐ I agree to participate in this survey.

Questions 1 to 5 are about yourself.

Question 1. How old are you currently? __ years old

Question 2. In which year did you graduate from junior college? (Heisei/Reiwa)

Question 3. Which major did you belong to?

a. Life Culture Major

b. Food and Nutrition Major

Question 4. Do you have any experience with pregnancy?

a. Yes

b. No (Please proceed to question 11.)

Question 5. In which year did you get pregnant? (If you have experienced multiple pregnancies, please answer the most recent year.) (Heisei/Reiwa)

Questions 6 to 20 are about folic acid.

Question 6. Did a doctor or healthcare professional provide you with information on taking folic acid supplements?

a. Yes

b. No

Question 7. During pregnancy, did you take folic acid supplements (excluding fortified rice)?

a. Yes

b. No

Question 8. When did you start taking folic acid supplements (excluding fortified rice)?

a. I started taking folic acid supplements more than a month before I knew I was pregnant.

b. I started taking folic acid supplements after I found out I was pregnant.

Question 9. During pregnancy, did you consume fortified rice (including folic acid)?

a. Yes

b. No

Question 10. When did you start consuming fortified rice (including folic acid)?

a. I started consuming fortified rice (including folic acid) more than a month before I knew I was pregnant.

b. I started consuming fortified rice (including folic acid) after I found out I was pregnant.

Please proceed to question 14.

Question 11. Have you been told by a doctor or healthcare professional to take folic acid supplements?

a. Yes

b. No

Question 12. Are you currently taking folic acid supplements (excluding fortified rice)?

a. Yes

b. No

Question 13. Are you currently consuming fortified rice (including folic acid)?

a. Yes

b. No

Question 14. Have you heard of the term "neural tube defects"?

a. Yes

b. No

Question 15. Do you know what is needed to reduce the risk of neural tube defects?

a. Yes

b. No

Question 16. Have you ever obtained information about folic acid?

a. Yes

b. No

Question 17. How did you obtain information about folic acid? (Multiple answers allowed)

a. TV

b. Internet

c. Magazines

d. Pamphlets created by the government or other organizations

e. Healthcare professionals

f. Others

Question 18. Do you remember learning about folic acid in your junior college classes?

a. Yes

b. No

Question 19. Have you ever provided information to others about the necessity of folic acid intake before pregnancy?

a. Yes

b. No

Question 20. Do you intend to take folic acid supplements before future pregnancies?

a. Yes

b. No

These are all the questions. Thank you very much for your cooperation.
